# Nanomedicine Strategies Against Biofilm‐Associated Infections: Advances, Challenges, and Translational Barriers

**DOI:** 10.1002/mbo3.70210

**Published:** 2025-12-28

**Authors:** Husni Farah, Munthar Kadhim‐Abosaoda, Hayjaa Mohaisen‐Mousa, S. Renuka Jyothi, Priya Priyadarshini‐Nayak, J. Bethanney Janney, Gurjant Singh, Ashish Singh‐Chauhan, Manoj Kumar‐Mishra

**Affiliations:** ^1^ Faculty of Allied Medical Sciences, Hourani Center for Applied Scientific Research Al‐Ahliyya Amman University Amman Jordan; ^2^ College of Pharmacy The Islamic University Najaf Iraq; ^3^ College of Pharmacy The Islamic University of Al Diwaniyah Al Diwaniyah Iraq; ^4^ Department of Medicinal Chemistry Al‐Turath University, Al‐Mansour Baghdad Iraq; ^5^ Department of Biotechnology and Genetics, School of Sciences JAIN (Deemed to be University) Bangalore Karnataka India; ^6^ Department of Medical Oncology, IMS and SUM Hospital Siksha ‘O’ Anusandhan (Deemed to be University) Bhubaneswar Odisha India; ^7^ Department of Biomedical Sathyabama Institute of Science and Technology Chennai Tamil Nadu India; ^8^ Department of Physiotherapy, University Institute of Allied Health Sciences Chandigarh University Chandigarh Punjab India; ^9^ Faculty of Engineering Gokul Global University Sidhpur Gujarat India; ^10^ Salale University Fitche Ethiopia

**Keywords:** antimicrobial resistance, biofilms, drug delivery, nanoparticles, translational nanomedicine

## Abstract

Antimicrobial resistance continues to rise globally, with biofilm‐associated infections intensifying the clinical burden through persistent tolerance to antibiotics and evasion of immune responses. Biofilms, structured microbial communities embedded in a protective extracellular matrix, underlie many chronic and recurrent infections, including endocarditis, urinary tract infections, cystic fibrosis lung disease, and device‐related infections. Conventional antibiotics often fail in these contexts, and the discovery pipeline for novel agents remains limited. Nanotechnology has therefore emerged as a promising alternative, offering unique physicochemical features that enable enhanced penetration into biofilm matrices, improved drug stability, and targeted delivery of therapeutic agents. Diverse nanosystems, including metallic, polymeric, lipid‐based, and ligand‐functionalized platforms, have shown encouraging results in vitro and in vivo, demonstrating superior biofilm disruption and bacterial eradication compared with conventional therapies. Nevertheless, translating these advances into clinical practice remains challenging. Key barriers include complex and costly synthesis, scalability under good manufacturing practices, limited drug loading efficiencies, variability of preclinical biofilm models, regulatory uncertainties, and the risks of nanoparticle (NP)‐induced toxicity, unpredictable biodistribution, and potential resistance development. Moreover, the dynamic interactions between NPs, host fluids, and biofilm extracellular matrices complicate pharmacokinetic and pharmacodynamic predictability. Addressing these obstacles requires coordinated efforts to refine manufacturing processes, standardize biofilm models, and implement nanospecific regulatory frameworks. With careful optimization, nanomedicine holds the potential to redefine the therapeutic landscape for biofilm‐related infections.

## Graphical abstract

Explore the key challenges and limitations in nanomedicine for biofilm treatment, from drug loading efficiency to antimicrobial resistance.

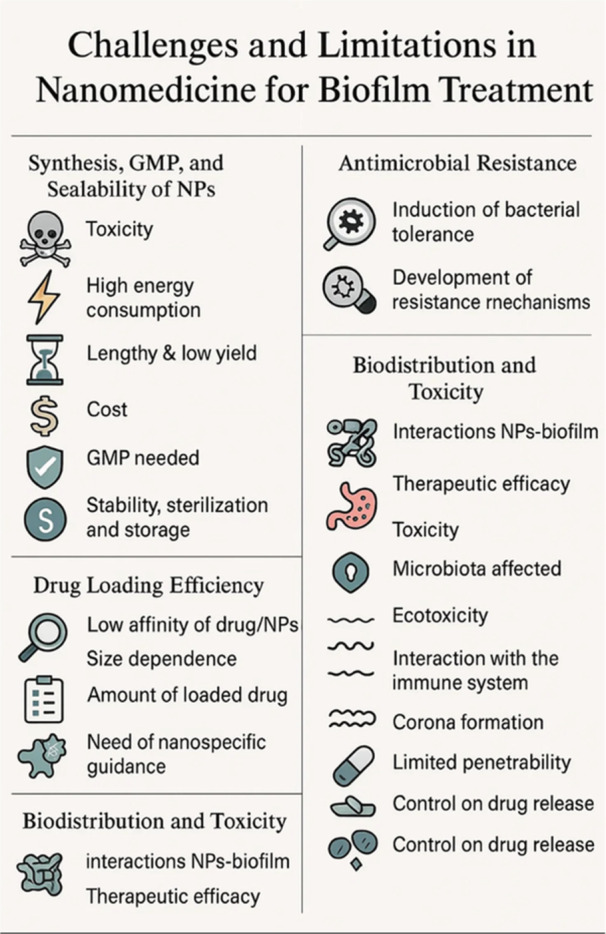

## 1 Introduction

Antimicrobial resistance (AMR) has emerged as one of the most pressing medical challenges of our time and is now formally recognized among the three greatest threats to public health by the European Commission's Health Emergency Preparedness and Response Authority. The global burden is alarming: in 2019 alone, nearly five million deaths were associated with bacterial resistance to antibiotics (Murray et al. 2022). In the United States, the Centers for Disease Control and Prevention estimates that every year more than 2.8 million resistant infections occur, with tens of thousands of lives lost as a consequence (Health UDo, Services H. 2019). AMR develops through natural genetic mutations and horizontal gene transfer, but its pace has been accelerated dramatically by inappropriate prescribing, overuse in humans and animals, and insufficient infection‐control practices (Salam et al. 2023). Compounding this problem is the remarkable ability of many microbes to organize themselves into biofilms, dense, structured microbial communities that provide a protective environment, drastically reducing susceptibility to antimicrobials and shielding them from the host immune response (Sharma et al. 2023).

Conventional drug development alone has not kept pace with this escalating threat. The pharmaceutical sector has deprioritized antibiotic discovery and production because of limited financial returns compared with other therapeutic markets (Almatroudi 2025). Despite ongoing initiatives in Europe, the United Kingdom, and the United States to stimulate antibacterial innovation through funding programs and policy incentives, the pipeline of new antibiotics remains sparse. This reality underlines the need for alternative therapeutic strategies (Årdal et al. 2020). Given that AMR and biofilm formation share overlapping survival mechanisms, such as efflux pump activation, horizontal gene transfer, and metabolic dormancy—addressing AMR effectively requires therapeutic systems capable of overcoming these biofilm‐associated defenses. This convergence positions nanomedicine as a complementary approach, capable of targeting biofilm‐associated defenses that conventional antibiotics alone cannot overcome (Uddin et al. 2021; Kumar et al. 2023).

One promising approach is the integration of nanotechnology into the study and treatment of microbial biofilms. Nanoparticles (NPs) have generated significant interest because of their unique physical and chemical properties. Their extremely small dimensions, typically ranging from a few nanometers to micrometers, allow them to infiltrate biofilm matrices more effectively than conventional agents, improving drug delivery and enhancing eradication (Kumar et al. 2023). In addition, the high surface area of NPs relative to their volume provides greater reactivity and enables efficient loading of active molecules, such as antibiotics, peptides, or enzymes. By encapsulating or attaching therapeutic compounds within nanosystems, pharmacokinetic and pharmacodynamic profiles can be optimized, solubility and stability enhanced, and degradation minimized, reducing systemic toxicity (Karahmet Sher et al. 2024). Furthermore, functionalization with specific ligands can direct NPs precisely toward biofilm sites, increasing local drug concentration and facilitating deeper penetration (Wang et al. 2017).

Nevertheless, translation from laboratory findings to bedside applications remains challenging. Considerable barriers exist, particularly the gap between simplified in vitro models and the complex nature of in vivo biofilm infections (Vyas et al. 2022). Despite extensive research into antimicrobial and antibiofilm agents, most existing therapies remain limited to either disrupting matrix integrity or inhibiting quorum sensing, and are largely ineffective against mature or polymicrobial biofilms in vivo. Current pharmacological and physical interventions fail to penetrate dense extracellular polymeric matrices, eliminate persister cells, or prevent rapid recolonization following treatment cessation. Hence, a substantial therapeutic gap persists between conventional antibiofilm strategies and the sophisticated defense mechanisms of biofilm communities (Sharma et al. 2023; Lan et al. 2025). Integrating nanomedicine approaches provides a rational framework to bridge this gap by enabling targeted drug delivery, enhanced biofilm penetration, and controlled release at infection sites. This review, therefore, aims to explore the potential of nanotechnology‐based antibiofilm therapies, highlight the progress achieved, and critically discuss the current obstacles limiting their clinical implementation.

## 2 Biofilms and Antibiofilm Therapies

Biofilms are not a rare phenomenon; they represent the dominant lifestyle of bacteria and archaea, accounting for most of the microbial life on Earth. Unlike their planktonic or free‐floating counterparts, microorganisms in biofilms acquire survival‐oriented traits that make them remarkably persistent (Flemming and Wuertz 2019). Within the clinical environment, this translates into heightened resistance to antimicrobials and evasion of immune defenses, making infections notoriously difficult to treat and eradicate (Chinemerem Nwobodo et al. 2022). As a result, biofilm‐associated infections contribute significantly to the chronicity and severity of diseases, such as infective endocarditis, urinary tract infections, cystic fibrosis lung disease, chronic wounds, inflammatory bowel disease, and chronic obstructive pulmonary disease, among many others (Vestby et al. 2020).

### 2.1 Biofilm Definition and Composition

A biofilm is essentially a three‐dimensional (3D) consortium of microorganisms adhered to one another and embedded within a self‐produced extracellular polymeric matrix. This matrix is typically composed of polysaccharides, proteins, extracellular DNA, and other substances such as lipids or cellulose, creating a robust scaffold that supports the microbial community (Karygianni et al. 2020). Its architecture is dynamic, influenced by environmental factors, such as temperature, pressure, ionic strength, and fluid shear. Depending on these conditions and the maturity of the biofilm, the extracellular matrix (ECM) can range from a loosely dispersed gel to a densely compact structure (Almatroudi 2025). Historically, biofilms were defined as microbial layers attached to biotic surfaces, such as teeth or mucosal tissue, or to abiotic medical devices like catheters (Sharma et al. 2023). However, more recent research recognizes that even nonattached microbial aggregates can function as biofilms. These suspended clusters have been implicated in respiratory infections with impaired clearance mechanisms and in persistent soft tissue infections (Cai 2020). Equally important is the recognition that biofilms are often polymicrobial rather than single species. In these mixed communities, different microorganisms interact, cooperate, or compete, often enhancing collective resilience against antimicrobial therapies and suppressing host immune responses. Such polymicrobial organization contributes not only to heightened tolerance but also to increased virulence and persistence of infections (Sharma et al. 2023).

### 2.2 The Biofilm Lifecycle

**Figure 1:**
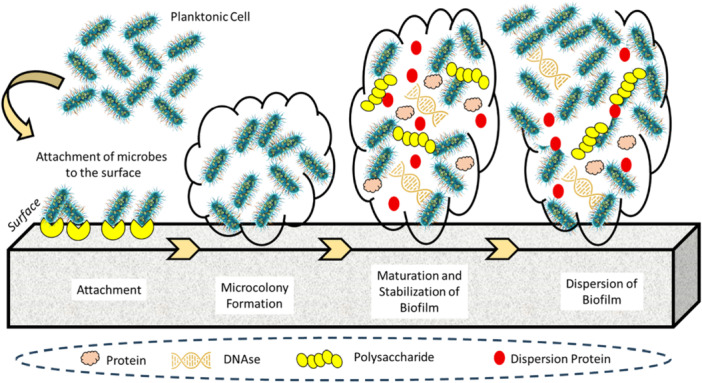
Schematic illustration of Biofilm cycle formation (Murray et al. 2022): initial reversible attachment of free‐floating planktonic bacteria to a surface, then irreversible attachment with production of extracellular polymeric substances (EPS) (Health UDo, Services H. 2019), early biofilm development and microcolony formation (Salam et al. 2023), biofilm maturation into a three‐dimensional structured community, and dispersion of cells back into the environment (Sharma et al. 2023). Use simplified shapes, arrows showing progression, and soft scientific colors (blue, teal, and green). DNAse, deoxyribonuclease.

The formation of a biofilm is not a random event but rather a tightly regulated process involving distinct developmental stages. Microbes undergo profound phenotypic shifts that alter their metabolic activity, gene expression, and structural organization depending on both species identity and surrounding conditions (Sauer et al. 2022). Although the diversity of biofilm forms is vast, researchers commonly describe the lifecycle in three general phases: initial aggregation and attachment to a surface or each other, subsequent growth and accumulation marked by matrix production and community expansion, and finally disaggregation or dispersion, during which cells are released to colonize new environments (Malešević and Jovčić 2025). This dynamic cycle ensures both stability within established niches and adaptability for spreading into fresh ecological or clinical sites (Ban‐Cucerzan et al. 2025). Figure 1 schematically illustrates these stages of biofilm development, highlighting the sequential transitions from attachment and microcolony formation to maturation and final dispersion. The figure visually emphasizes how microbial communities evolve from initial surface contact into a highly organized and resilient 3D structure before dispersing to colonize new environments.

The initial step in biofilm development, known as aggregation and attachment, begins when free‐floating (planktonic) microorganisms encounter a surface or, in the case of nonsurface‐associated biofilms, attach directly to one another to form small clusters. This seemingly simple contact sets in motion a profound cellular transformation (Sharma et al. 2023). During this phase, microbes undergo extensive transcriptional reprogramming, halting motility driven by flagella, switching on the production of exopolysaccharides that serve as the structural backbone of the extracellular biofilm matrix, and activating genes linked with AMR (Singh et al. 2021). Once anchored, the community expands and evolves into a mature, 3D architecture. Within this structure, sharp gradients of oxygen, nutrients, waste products, and signaling molecules arise. These gradients foster the emergence of distinct microbial subpopulations, each adapting its gene expression to local microenvironments (Gallardo‐Navarro et al. 2024). Among the most clinically relevant of these subgroups are “persister cells.” These cells divide very slowly or not at all, rendering them highly tolerant to antimicrobial therapies. Importantly, they act as a reservoir capable of reseeding the biofilm once treatment is discontinued, thereby sustaining chronic infections (Moldoveanu et al. 2021). The final stage of the cycle, referred to as disaggregation and dispersion, involves the detachment of either individual planktonic cells or larger fragments of the mature biofilm. These released elements disseminate to new locations, closing the biofilm lifecycle and initiating colonization elsewhere (Sauer et al. 2022). This dynamic process ensures not only persistence within established niches but also the continual spread of infection to new host tissues or medical devices (Almatroudi 2025).

### 2.3 Biofilm Detection and Study: In Vitro and In Vivo Models

Although professional societies such as the European Society of Clinical Microbiology and Infectious Diseases (ESCMID) and the Infectious Diseases Society of America (IDSA) regularly publish expert guidelines, there is still no universal clinical standard for diagnosing biofilm‐related infections. In practice, detection remains a formidable challenge (Paul et al. 2022). Many biofilm‐associated infections progress silently for extended periods, only becoming apparent when bloodstream involvement occurs. When biofilms colonize implanted devices, they are often only identifiable after surgical removal, and even then, isolating viable microbial cells from the dense matrix can be technically demanding (Khatoon et al. 2018). Furthermore, some microbes are notoriously difficult to culture under laboratory conditions, leading to false‐negative results in routine diagnostic tests. An additional complication is that antimicrobial susceptibility testing is almost always performed on planktonic bacterial cultures, which significantly underestimates the level of resistance observed in true biofilm‐associated infections (Tetz and Tetz 2022).

To overcome these barriers, a broad array of laboratory models has been developed. In vitro systems provide simplified yet versatile platforms for cultivating biofilms from clinical isolates and for screening potential therapeutic agents. The most common of these are static models, such as microtiter plate assays or the Calgary Biofilm Device (Cometta et al. 2024). They are low‐cost, allow simultaneous testing of multiple strains and conditions, and are technically straightforward. However, their major drawback is that they poorly replicate the dynamic environment of an actual infection (Shi et al. 2019). By contrast, dynamic models such as flow chambers or the Robbins device better simulate in vivo conditions by incorporating nutrient flow and shear stress. Although more technically demanding and lower throughput, these systems provide more physiologically relevant data (Guzmán‐Soto et al. 2021). Recent advances have given rise to microfluidic‐based platforms, which miniaturize dynamic biofilm culture systems. These devices, such as the Bioflux system or the BiofilmChip, reduce reagent consumption, offer high experimental versatility, and are relatively easy to fabricate (Wen et al. 2024). Microfluidics also allow fine control over fluid dynamics and spatial gradients, providing a closer approximation of natural biofilm conditions while remaining adaptable for high‐throughput screening (Harvey et al. 2022).

Animal models remain indispensable for validating laboratory findings and for elucidating the host's contribution to infection dynamics. A wide variety of species and procedures have been used, complicating direct comparison across studies, but certain disease‐specific models have become well established (Domínguez‐Oliva et al. 2023). For example, rodent models are frequently used for chronic otitis media, nonhealing wounds, rhinosinusitis, and osteomyelitis. These in vivo systems are particularly valuable for assessing immune responses, testing antimicrobial strategies in a physiologically relevant context, and bridging the gap between experimental research and clinical translation (Bahamondez‐Canas et al. 2019).

### 2.4 General Overview of Biofilm Treatment Strategies

The biofilm growth mode presents a formidable therapeutic challenge. Unlike free‐living bacteria, biofilm‐embedded cells display multiple protective mechanisms that render traditional antimicrobials far less effective. The ECM itself impedes penetration of antibiotics and disinfectants, reducing drug access to the deepest layers (Sharma et al. 2023). In addition, metabolic gradients within the biofilm create heterogeneous microenvironments: cells near the surface may be actively dividing, while those in deeper layers often adopt a slow‐growing or dormant state, making them intrinsically less responsive to antimicrobial attack (Lan et al. 2025). Other defense mechanisms include modifications of the cell envelope, altered enzyme production, and the upregulation of multidrug efflux pumps that actively expel therapeutic compounds (Uddin et al. 2021). Horizontal gene transfer further accelerates the spread of resistance traits within the biofilm community. Taken together, these characteristics explain why biofilm‐associated infections are so persistent and why conventional therapies frequently fail to achieve complete eradication (Michaelis and Grohmann 2023).

In light of these obstacles, the medical and scientific communities are actively exploring alternative or complementary therapeutic approaches. Strategies under investigation range from agents that disrupt the biofilm matrix, to molecules that inhibit quorum sensing, to innovative delivery platforms such as NPs that enhance antimicrobial penetration (Lan et al. 2025). Physical and mechanical methods, including ultrasound, photodynamic therapy, and electrical stimulation, are also being studied as adjunctive approaches (Karkhaneh et al. 2025). The following sections of this review aim to provide a clear overview of these therapeutic strategies, classified according to their principal mechanisms of action, while situating them within the broader landscape of biofilm management (Table 1).

**Table 1:** Key aspects of biofilms and therapeutic strategies for their management.

Aspect	Description	Clinical relevance	Limitations/challenges	Examples/models/applications	Reference
Definition and composition	Biofilms are structured microbial consortia embedded in an extracellular polymeric matrix (polysaccharides, proteins, eDNA, and lipids).	Provide structural support and resilience against host defenses and antimicrobials.	ECM composition varies by species, maturity, and environment, complicating treatment.	Dental plaque, catheter biofilms, and respiratory tract aggregates.	Perry and Tan (2023)
Lifecycle	Three main stages: (i) aggregation/attachment, (ii) growth and accumulation, and (iii) disaggregation/dispersion.	Explains persistence and recurrence of infections; persister cells maintain infection after therapy.	Strong phenotypic heterogeneity; resistant subpopulations emerge.	*Pseudomonas aeruginosa* in cystic fibrosis lung biofilms.	Sauer et al. (2022)
Detection and study	Diagnostic challenges: Standard tests target planktonic cells; biofilms are often undetected until advanced.	Critical for guiding therapy; conventional susceptibility tests underestimate resistance.	Difficult to culture; no standardized clinical protocols.	In vitro: Microtiter plates, Calgary device. In vivo: Rodent wound/otitis media models.	Magana et al. (2018)
Treatment: Physical removal	Direct elimination of biofilm mass by surgery, irrigation, or debridement.	Standard in device‐associated infections; enhances antibiotic efficacy.	Highly invasive; site accessibility dependent; not always feasible.	Catheter removal, wound irrigation with jets.	Y. Su, Yrastorza, et al. (2022)
Treatment: Structural disruption	Target ECM using enzymes, chelators, or polysaccharide analogs to destabilize matrix.	Enhances penetration of antimicrobials; increases bacterial susceptibility.	ECM heterogeneity; enzyme specificity requires pathogen identification.	Dispersin B, Pulmozyme, phage‐derived depolymerases.	Kalia et al. (2023)
Treatment: Antimicrobial agents	Direct killing via natural/synthetic compounds or stress‐inducing therapies.	Potential to eradicate biofilm biomass.	High concentrations are often needed; systemic toxicity risks.	Antibiotic–nitroxide hybrids, photodynamic therapy, phage cocktails (e.g., PYO).	Lan et al. (2025)
Treatment: Quorum sensing and stress response inhibition	Block microbial communication or stringent response to impair biofilm maintenance.	Leads to detachment and collapse of biofilms; reduces persistence.	Complex regulation; species‐specific responses; resistance may develop.	Quorum‐sensing inhibitors, ppGpp pathway blockers.	Almatroudi (2025)
Treatment: Drug delivery systems	Nanoparticles (NPs), liposomes, and micelles are engineered for targeted delivery and controlled release.	Improve penetration, reduce systemic toxicity, and sustain therapeutic concentrations.	Cost, stability, regulatory approval, and scale‐up limitations.	PLGA NPs, BiofilmChip models, functionalized liposomes	Blanco‐Cabra et al. (2024)

Abbreviations: ECM, extracellular matrix; eDNA, environmental DNA; PLGA, poly(lactic‐*co*‐glycolic acid); ppGpp, guanosine tetraphosphate; PYO, pyocyanin.

#### 2.4.1 Physical Removal of Biofilms

The most straightforward and historically oldest method of managing biofilm infections is their direct physical elimination. This strategy is particularly relevant in infections linked to medical devices, where removal of the contaminated implant or catheter is often the first and most effective step before initiating antimicrobial therapy. In such cases, eradicating the foreign body dramatically reduces microbial burden and enhances the efficacy of antibiotics (Kumar et al. 2023). Similarly, in conditions such as oral biofilms, infected wounds, or joint infections, clinical management frequently incorporates irrigation with water jets or surgical debridement to mechanically reduce biofilm biomass prior to pharmacological treatment. However, the practicality of this approach is highly case‐dependent, as its success is dictated by the accessibility of the infected site, the invasiveness of the intervention, and the overall health of the patient (Y. Su, Yrastorza, et al. 2022).

#### 2.4.2 Structural Disruption of Biofilms

Recognizing the limitations of purely mechanical approaches, strategies have been developed to directly compromise the integrity of biofilm architecture. By targeting the ECM, the protective, self‐produced scaffold surrounding the microbial community, it is possible to weaken biofilm stability, enhance detachment, and render embedded bacteria more vulnerable to antimicrobials (Lu et al. 2024). Among the most promising agents are enzymes that specifically degrade matrix components, including those derived from bacteriophages. Laboratory studies have demonstrated their ability to dismantle biofilms effectively at relatively low concentrations, with the added advantage of reduced risk of resistance development compared with conventional antibiotics (Almatroudi 2025).

Nevertheless, a key challenge lies in the variability of ECM composition, which differs according to microbial strain, environmental context, and biofilm maturity. The high specificity of phage‐derived enzymes, while beneficial, also limits their applicability unless the causative organism is known in advance (Coppola et al. 2025). To address these issues, researchers are exploring combined approaches, such as cocktails of enzymes targeting multiple matrix components, or pairing enzymatic therapies with conventional antibiotics, especially in polymicrobial infections where complexity is greater (Poilvache et al. 2021). Some enzyme‐based therapies, such as dispersin B and Pulmozyme, have progressed into clinical use, though safety concerns and potential cytotoxicity remain barriers for broader application (Kalia et al. 2023). In addition to enzymes, other agents such as exopolysaccharides and calcium‐chelating molecules can destabilize the ECM by disrupting ionic interactions and inducing biofilm dispersion, thereby improving drug access and overall treatment efficacy (Sharma et al. 2023).

#### 2.4.3 Antimicrobial Agents

A second therapeutic approach focuses on reducing biofilm biomass by directly inducing microbial death. Importantly, the antibacterial potency of an agent in planktonic cultures does not guarantee equal success against biofilm‐embedded bacteria. Still, a number of natural and synthetic compounds have been shown to exert antibiofilm activity, some originating from plants or microbial secondary metabolites (Lu et al. 2024). Novel strategies also include stress‐inducing therapies that promote intracellular accumulation of reactive oxygen and nitrogen species. Examples include antibiotic–nitroxide hybrids, photodynamic therapy, electrical or voltage‐based stimulation, and plasma‐based technologies coupled with water electrosprays. These interventions disrupt microbial homeostasis and exert cytotoxic effects deep within biofilms (Jiang et al. 2025).

In addition, drug repurposing efforts have uncovered unexpected antibiofilm properties in agents originally designed for other therapeutic areas, including oncology drugs (Z. Zhang et al. 2020). While some of these agents show impressive efficacy in eradicating biofilms, their effective concentrations are often significantly higher than those approved for their primary indications, creating translational barriers (Verderosa et al. 2019). Biological strategies such as bacteriophage therapy and the use of predatory bacteria offer alternative avenues. Phages can selectively infect and lyse biofilm‐forming bacteria, while predatory microbes can directly attack pathogenic species (Visnapuu et al. 2022). Phage cocktails such as pyocyanin, developed for chronic wound infections, have already reached commercial availability. However, the use of predatory bacteria remains experimental, with concerns over unintended impacts on the host microbiota limiting clinical progression (Mayorga‐Ramos et al. 2024).

#### 2.4.4 Inhibition of Quorum Sensing and Stringent Response

Beyond physically dismantling biofilms or directly killing their inhabitants, another promising therapeutic axis is interference with bacterial communication and stress‐response networks (Muhammad et al. 2020). Microbes within biofilms coordinate their behavior through quorum sensing, a sophisticated signaling system that regulates swarming, population density, and virulence factor production. Disrupting these signals prevents the synchronized behaviors necessary for maintaining biofilm structure, often leading to detachment and collapse of the microbial community (Preda and Săndulescu 2019). Similarly, targeting the stringent response, the adaptive mechanism by which bacteria reprogram gene expression during nutrient deprivation, represents another opportunity. Since the stringent response is essential for maintaining biofilm homeostasis and persistence, its inhibition may weaken biofilm resilience and improve antimicrobial susceptibility (Urwin et al. 2024).

#### 2.4.5 Advanced Drug Delivery Systems

Beyond discovering new antibiofilm molecules, significant research efforts focus on optimizing how therapeutic agents are delivered. Advanced drug delivery systems aim to enhance stability, bioavailability, safety, and controlled release, thereby improving treatment outcomes while minimizing toxicity (Muteeb et al. 2023). Nanotechnology has emerged as a leading approach in this field. Due to their small size, NPs can penetrate deeply into biofilm matrices through pores that conventional drugs cannot easily traverse. Their high surface area also allows efficient drug loading and the possibility of functionalization with targeting ligands (Mitchell et al. 2021).

**Figure 2:**
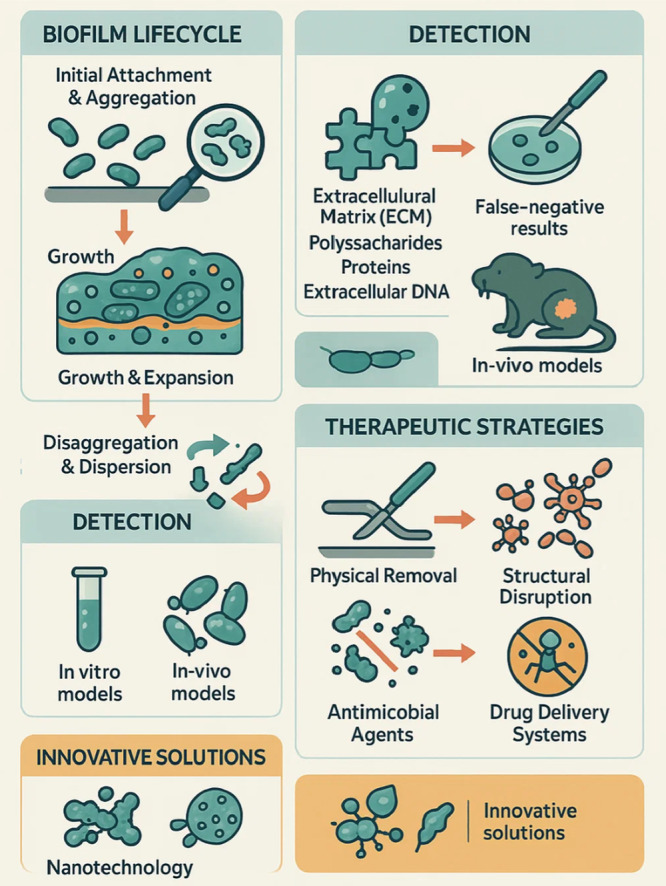
Exploring the complex lifecycle of biofilms and the persistent challenges they pose in treating chronic infections. Innovative strategies, including nanotechnology and antimicrobial therapies, offer hope for effective eradication.

These nanosystems are versatile, minimally invasive, and capable of achieving precise, localized drug release, reducing the likelihood of resistance development. Despite their promise, only a limited number have advanced to clinical application (Islam et al. 2025). Substantial translational challenges, such as large‐scale production, long‐term safety, and regulatory approval, must still be resolved. The persistent tolerance of biofilms to antibiotics and host immunity highlights the necessity of strategies that act at both physical and molecular levels. Nanomedicine directly addresses these barriers through its capacity to penetrate the biofilm matrix, deliver drugs in a controlled manner, and interact selectively with microbial cells. By bridging the mechanical protection of the ECM and the metabolic dormancy of persister cells, nanosystems provide a mechanistic link between conventional antibiofilm concepts and modern precision drug delivery (Lan et al. 2025; Fulaz et al. 2019). Hence, nanotechnology represents not merely a new formulation platform, but a fundamentally distinct paradigm capable of overcoming core biofilm defense mechanisms. The following section will explore in detail the major nanotechnology‐based strategies for antibiofilm therapy, highlighting both their potential and their limitations in real‐world clinical contexts (Campos et al. 2025) (Figure 2).

## 3 Nanomedicines for Biofilm Treatment: Challenges and Limitations

In the past decade, nanotechnology has become one of the most actively explored strategies for combating biofilm‐associated infections. A growing body of literature describes a wide spectrum of NPs with diverse compositions, properties, and mechanisms of action (Kumar et al. 2023). Comprehensive reviews have systematically classified these nanomedicines, examining their physicochemical features, therapeutic potential, and the barriers to clinical translation (Blanco‐Cabra et al. 2024). Although nanotechnology offers significant advantages for antibiofilm therapy, its clinical translation remains limited. Several drawbacks persist, including potential cytotoxicity to mammalian cells, unpredictable biodistribution, aggregation in physiological fluids, and challenges in ensuring uniform drug release profiles. Additionally, large‐scale manufacturing often leads to batch variability and stability loss, while the regulatory landscape for nanomedicine approval is still fragmented across regions (Ma et al. 2024; Blanco‐Cabra et al. 2024). Hence, the benefits of NPs should be viewed as conditional upon addressing these physicochemical and translational constraints.

Specific categories include organic NPs, polymer‐based carriers, lipid systems, and inorganic or metallic particles. Other works have analyzed delivery‐oriented strategies, such as encapsulation, surface modification, and ligand functionalization, which allow more precise targeting of biofilms while improving pharmacokinetics (Campos et al. 2025). Some reviews also emphasize the dynamic interplay between NPs and the biofilm ECM, highlighting how surface chemistry and charge determine penetration and efficacy (Fulaz et al. 2019). To illustrate real‐world applications, several studies employing robust in vitro and in vivo models are noteworthy. For instance, iron oxide (Fe_3_O_4_) magnetic NPs have been used against periodontal biofilms, with magnetic targeting enhancing penetration and antibacterial efficacy in rat models of gum inflammation (Tong et al. 2023). In another study, cationic liposomes carrying enzymes such as DNase I and Proteinase K effectively disrupted *Cutibacterium acnes* biofilms in skin and catheter‐associated infections (Fang et al. 2021). Similarly, lipid‐based micellar nanocarriers responsive to acidic and hypoxic conditions were loaded with antibiotics to treat *Staphylococcus aureus* biofilms in vivo (L. Su, Li, et al. 2022).

Innovative polymeric nanoplatforms have also been developed. One example includes dendrimer‐conjugated azithromycin NPs capable of adapting their size and charge to penetrate *Pseudomonas aeruginosa* lung biofilms, disassembling upon arrival to maximize intracellular antibiotic delivery (Shariati et al. 2022). Functionalized poly(lactic‐*co*‐glycolic acid) (PLGA) NPs carrying antistaphylococcal antibodies have shown promise in skin infection models, while dextran‐based NPs have been designed to enhance the penetration of tobramycin into *Pseudomonas* biofilms by neutralizing its charge (Miranda Calderon et al. 2023). These advances demonstrate the vast potential of nanomedicine in biofilm management. However, they also underscore the complexity of translating benchtop innovation into safe, effective, and widely accessible clinical therapies. Issues such as cytotoxicity, biodistribution, immunogenicity, large‐scale manufacturing, and cost‐effectiveness remain critical hurdles (Kazi et al. 2025).

### 3.1 Synthesis, Good Manufacturing Practices (GMPs), and Scalability of NPs

Methods used to produce nanomedicines (particularly metal‐based NPs) raise practical, environmental, and safety concerns. Conventional physical and chemical syntheses often demand high energy inputs and employ organic solvents or reactive reagents, creating potential hazards for workers and ecosystems (Pan et al. 2025). In addition, precious‐metal formulations such as silver and gold NPs can be markedly more expensive to manufacture than alternatives based on copper, zinc, titanium, or nonmetallic carriers, which complicates cost–benefit calculations during development (Yaqoob et al. 2020).

“Green” routes to NP production have been advanced as a partial remedy. Using fungi, bacteria, algae, or botanical extracts can reduce the reliance on harsh chemicals, improve biocompatibility profiles, and lower energy consumption while generating fewer toxic byproducts. These advantages, however, come with trade‐offs (Abuzeid et al. 2023). Biological feedstocks may be seasonal or geographically limited, some biosynthetic steps proceed slowly, and the underlying mechanisms of biosynthesis are not always fully understood (Joudeh and Linke 2022). Yields may be modest, and batch‐to‐batch variability in particle size and polydispersity remains a recurring problem that complicates downstream quality control (Mülhopt et al. 2018). Even when a promising laboratory protocol exists, scaling it for medical use is a major hurdle. Manufacturing must comply with GMPs, which are essential for reproducibility and patient safety but substantially increase costs (Ma et al. 2024). Sterilization and storage pose particular challenges: commonly used sterilization procedures can alter key NP attributes, surface chemistry, release kinetics, colloidal stability, or inadvertently increase toxicity. Because nanomedicines are structurally diverse, each formulation typically requires its own validated sterilization strategy (Harish et al. 2022). Stability during storage is another bottleneck. While researchers frequently use freshly prepared materials, commercial products must remain stable for months. Lipid‐based systems (e.g., liposomes) are prone to leakage and degradation over time, whereas many polymeric carriers show better shelf stability (Mehta et al. 2023). For bio‐derived or “green” metals, long‐term stability depends heavily on the exact synthesis route and capping agents employed (Mohammadi Dargah et al. 2024).

**Figure 3:**
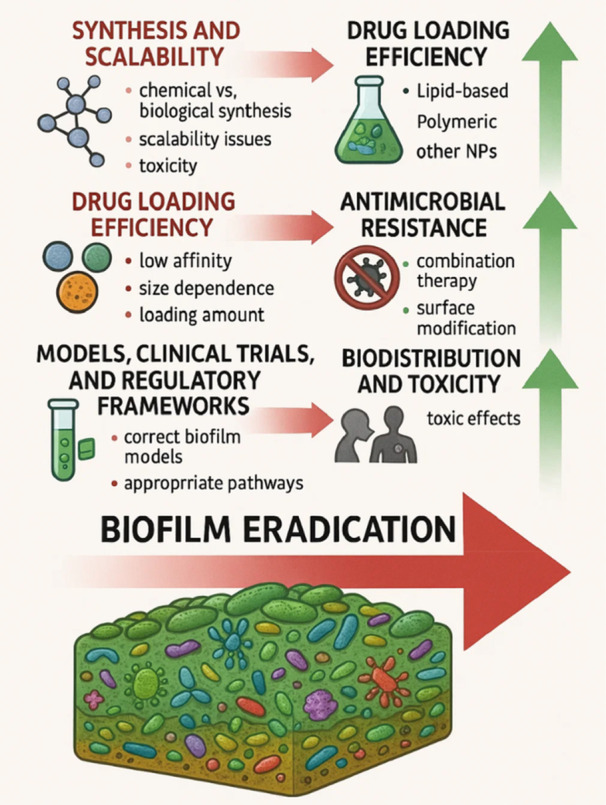
Overcoming challenges in nanomedicine to combat biofilm infections. A roadmap to advancing therapeutic strategies and ensuring biofilm eradication. (Generated with the assistance of ChatGPT, OpenAI, october 2025)

These realities force developers to weigh cost‐effectiveness at every step. Process intensification tools, such as carefully optimized lyophilization, can extend shelf life, but must be engineered meticulously to prevent aggregation, preserve particle size and drug release profiles, and avoid loss of biological activity. In short, moving from bench to bedside requires not only clever chemistry, but also rigorous process engineering, robust analytical characterization, and realistic economic planning (Yusuf et al. 2023) (Figure 3).

### 3.2 Drug Loading Efficiency

Encapsulating antibiotics within nanosystems can shield labile drugs from degradation, reduce off‐target toxicity, and enable more controlled, prolonged release at the site of infection. Two related but distinct efficiency metrics govern whether a platform is practical (Yeh et al. 2020). Encapsulation efficiency describes the fraction of the input drug that ends up associated with the carrier; this parameter largely hinges on the fabrication method and determines process waste and scalability (Bi et al. 2025). Drug loading (or loading capacity) quantifies the mass of drug per unit mass of carrier; this value directly influences antimicrobial potency for a given administered dose and depends on the chemistry of both payload and vehicle (Yusuf et al. 2023).

Several design tensions emerge. Hydrophilic drugs can be difficult to entrap within hydrophobic matrices, and some widely used materials, such as certain grades of PLGA, may achieve acceptable encapsulation but still provide relatively low loading capacities, forcing administration of larger quantities of carrier to reach therapeutic exposure (Alsaab et al. 2022). In anatomical sites with strict dosing limits (e.g., the lungs), high loading becomes essential; yet many studies report encapsulation data without providing the equally critical loading values, making clinical translation difficult to judge (Ma et al. 2024). A related trade‐off involves particle size. Increasing particle diameter generally increases the absolute amount of drug that can be carried. However, pushing formulations into the submicron range to boost loading can compromise penetration through dense biofilm matrices (Heydari et al. 2024). Effective product design, therefore, balances sufficient loading for therapeutic effect with particle sizes small enough to traverse the extracellular polymeric substance and reach deeply embedded bacteria (Kayoumu et al. 2025).

### 3.3 In Vitro and In Vivo Biofilm Models, Clinical Trials, and Regulation

Recreating the biological reality of biofilm infections for testing is inherently challenging. Planktonic assays, though convenient for measuring conventional antibiotic susceptibility, do not reflect the physiology of biofilm‐embedded cells (Sharma et al. 2023). Static biofilm models provide a first approximation of antibiofilm activity, yet they cannot capture the fluid shear, nutrient gradients, and evolving microenvironments that characterize infections in vivo (Y. Su, Yrastorza, et al. 2022). Dynamic systems address these gaps by introducing flow and controlled gradients, enabling more realistic assessments of penetration, pharmacokinetics, and pharmacodynamics. Ex vivo models using human tissues, when ethically permissible, add another layer of relevance by preserving native architecture and extracellular components (Desai et al. 2025).

Nevertheless, none of these platforms alone can fully predict the behavior of a chronic infection in a patient. Animal models remain indispensable for understanding whole‐organism pharmacology, distribution, immune interactions, and toxicity. Selecting the right model demands alignment between pathogen, disease site, and host species, which is not always feasible (Ma et al. 2024). Some human pathogens colonize animals poorly; others evolve differently in the presence of animal immune systems. Moreover, chronic biofilm infections in humans often unfold over months or years, whereas practical constraints limit many in vivo experiments to days or a few weeks (Nesse et al. 2023). Polymicrobial infections add further complexity: coculturing multiple species in stable communities is technically difficult, even though such consortia are common in clinical settings. Prioritizing clinical isolates and resistant strains over laboratory workhorses can improve translational value but increases experimental complexity (Srinivasan et al. 2023).

These scientific hurdles intersect with regulatory ones. Despite encouraging in vitro results for many nano‐enabled antimicrobials, relatively few have reached clinical trials. Evolving or heterogeneous regulatory frameworks can slow progress, particularly when small changes in polymer chemistry or surface functionalization create “new” products that require additional safety and efficacy data (Desai et al. 2025). Clearer, nanomedicine‐specific guidance, covering standardized characterization, biocompatibility, and nanotoxicology, would help streamline the path from promising preclinical data to human studies (Ma et al. 2024). To provide a quantitative and visual overview of the current status of nanomedicine‐based antibiofilm research, Table 2 summarizes the number of studies at each developmental stage, representative examples, and key limitations.

**Table 2:** Quantitative overview of nanomedicine‐based antibiofilm research and clinical translation (as of 2025).

Development stage	Typical study focus	Approximate number of studies	Representative examples	Key limitations
In vitro models	Screening antibiofilm activity of nanoparticles (NPs); characterization of penetration and killing efficiency	> 300	Metallic, polymeric, lipid‐based, and hybrid NPs tested against *Staphylococcus aureus*, *Pseudomonas aeruginosa*, and *Escherichia coli*	Simplified biofilm structure; poor physiological relevance
In vivo (animal) studies	Testing efficacy and toxicity in rodent models of wound, lung, and implant infections	~ 60–80	Magnetic Fe_3_O_4_ NPs, cationic liposomes, PLGA‐based carriers	Short duration; limited chronicity; interspecies variability
Preclinical/translational	Scalable formulation, GMP synthesis, and regulatory compliance studies	~ 15–20	Dendrimer–antibiotic conjugates, antibody‐functionalized PLGA NPs	High cost, manufacturing variability
Clinical evaluation	Early‐stage human trials or compassionate use for chronic wound and device‐related infections	< 5	Silver‐based nanosystems and lipidic nanocarriers	Safety, biodistribution, and regulatory hurdles

Abbreviations: GMP, good manufacturing practice; PLGA, poly(lactic‐*co*‐glycolic acid).

As summarized in Table 2, many studies remain confined to in vitro investigations, with comparatively few progressing to animal models or translational stages. Fewer than five nanomedicine‐based formulations have reached early clinical testing, highlighted critical translational bottlenecks and underscored the urgent need for standardized preclinical models, scalable manufacturing protocols, and harmonized regulatory frameworks to accelerate clinical adoption.

### 3.4 AMR in the Context of NPs

A frequently raised concern is whether widespread use of nanomaterials could select for new resistance phenotypes. NPs can influence bacterial physiology by altering membrane permeability, modulating efflux systems, and imposing oxidative or osmotic stress; in principle, adaptive responses to these pressures might spill over to affect susceptibility to traditional antibiotics (Gupta et al. 2019). Reports of reduced sensitivity to certain nanomaterials suggest that selection is possible, and tolerance phenomena, such as NP‐induced dormancy within biofilms, could further blunt antimicrobial efficacy (Almatroudi 2025).

At present, NP resistance does not appear to be prevalent, but environmental exposure is rising as nano‐enabled products enter soil and water systems that already serve as reservoirs for antibiotic‐resistant organisms. Prudent stewardship is therefore warranted (Muteeb et al. 2023). Careful materials selection, dose optimization, and environmental risk assessment should accompany the clinical development of nano‐based antibiofilm therapies to minimize unintended ecological and evolutionary consequences (Parvin et al. 2025).

### 3.5 Biodistribution and Toxicity of NPs

A thorough understanding of how NPs distribute, persist, and clear from the body is fundamental to safe clinical use. Biodistribution profiles depend on administration route and formulation, and they shape both therapeutic index and adverse‐event risk (Kumar et al. 2023).

#### 3.5.1 Interactions with Biofilms: Protein Corona and Controlled Release

Before NPs can act on a biofilm, they must reach the infected niche. The journey, whether via systemic administration, inhalation, topical delivery, or local injection, exposes them to complex biological fluids rich in proteins, lipids, polysaccharides, metabolites, and nucleic acids (Guo et al. 2023). These molecules rapidly adsorb to the particle surface to form a protein (biomolecular) corona, which evolves over time from a loosely bound “soft” layer to a more stable “hard” layer of higher‐affinity constituents. The corona strongly influences cellular recognition, immune interactions, and trafficking (Abarca‐Cabrera et al. 2021). Once at the infection site, additional components from the biofilm matrix can further remodel the corona, altering particle–microbe interactions in ways that may not be predicted by in vitro testing (Sharma et al. 2023).

Penetrating the biofilm's ECM is the next barrier. Surface engineering with targeting ligands, such as antibodies, lectins, or antimicrobial peptides, can enhance adhesion to bacterial cells and improve passage through the matrix. Yet the corona formed en route can mask or modify these ligands, partly explaining discrepancies between benchtop efficacy and in vivo performance (Scalia and Najmi 2025). Upon successful penetration, therapeutic success depends on delivering the payload in a controlled manner. Ideal profiles often entail an initial burst to reduce biomass, followed by sustained concentrations above the minimum inhibitory level to prevent regrowth from persister populations (Le et al. 2021). Different carrier classes excel in different aspects: polymeric systems frequently offer superior control over release kinetics but may have lower loading capacities, whereas liposomal formulations can carry more drug but sometimes release it too quickly (Floyd et al. 2025). Externally triggered systems, responsive to magnetic fields, temperature, light, or ultrasound, or internally triggered designs, responsive to pH, enzymes, or redox conditions, provide additional levers to synchronize drug release with the biofilm microenvironment (Liu et al. 2019).

#### 3.5.2 Clearance and Toxicity

Clearance kinetics must balance safety and efficacy. Rapid elimination can reduce systemic toxicity but may curtail exposure at the infection site; slow clearance can improve efficacy but raises concerns about accumulation (Desai et al. 2024). Particle size is a primary determinant: very small NPs may be filtered by the kidneys and excreted in urine; intermediate sizes are often sequestered by hepatic Kupffer cells and processed in the liver; larger particles tend to be removed by splenic macrophages, while the largest formulations may circulate longer but risk uptake by the reticuloendothelial system (Dogra et al. 2020). Surface properties and the composition of the protein corona modulate these pathways by promoting opsonization and complement activation, potentially leading to premature clearance (Panico et al. 2022).

Toxicity is multifactorial, influenced by composition, dose, size and shape, surface charge, stability, and route of administration. Metallic NPs frequently rely on reactive oxygen species generation for antimicrobial action, a mechanism that can also harm host tissues via oxidative stress (Skłodowski et al. 2023). Photothermal and magnetothermal approaches harness local hyperthermia to suppress bacteria, but require precise control to avoid collateral injury (Huo et al. 2021). Even biodegradable polymers such as chitosan, alginate, PLGA, or PVA can accumulate or cause local irritation if degradation is slow or incomplete (Lee and Kim 2025). Finally, broad antimicrobial activity risks disturbing commensal microbiota, with downstream consequences, such as dysbiosis (Lin et al. 2021).

Environmental considerations are also relevant. NPs released into air, soil, and water can interact with microbial communities and aquatic food webs, with uncertain long‐term ecological effects. These risks reinforce the need for comprehensive safety evaluations spanning product lifecycles—from manufacturing through clinical use to disposal. Collectively, these issues argue for rigorous, early toxicology and risk–benefit assessments tailored to nanomedicines (Ma et al. 2024). Failures in late‐stage development have sometimes stemmed from unforeseen corona effects that altered drug release or triggered immune reactions. Selecting appropriate cellular models and refining in vitro toxicology protocols can improve the prediction of in vivo outcomes and reduce attrition later in development (Table 3) (Waheed et al. 2022).

**Table 3:** Key challenges for nano‐enabled antibiofilm therapies and actionable mitigation paths.

Domain	Specific challenge	Mechanistic/practical impact	Typical evidence/models	Mitigation/best practices	Illustrative examples	Reference
Synthesis and GMP scale‐up	Energy‐/solvent‐intensive syntheses (especially metals)	Environmental/safety burden; batch variability	Bench protocols; pilot batches	Shift to green/biogenic routes; solvent minimization; QbD/DoE optimization	Ag/Au versus Cu/Zn/Ti NPs; biogenic AgNPs	Abuzeid et al. (2023)
	High cost of precious metals	Weak cost‐effectiveness at scale	Technoeconomic assessments	Material substitution; core–shell designs to reduce noble metal content	Au‐core/thin‐shell hybrids	Yaraki et al. (2022)
	Sterilization alters properties	Changes to size/charge/release; toxicity drift	Post‐sterilization analytics	Validate method per product (filtration/γ/Et0); protectants/lyoprotectants	Liposomes sensitive to γ‐irradiation	Morin et al. (2024)
	Storage instability	Leakage/aggregation; loss of potency	Real‐time/accelerated stability	Optimized lyophilization; cryo/lyoprotectants; robust caps	Liposomes (leakage) versus polymers (stable)	Narayanan et al. (2022)
Drug loading efficiency	Low loading (e.g., PLGA with hydrophilic drugs)	High carrier dose needed; clinical impracticality	In vitro loading/EE% reports	Chemistry matching (ion‐pairing, prodrugs); porous matrices; cosolvents	PLGA–antibiotic systems; ion‐paired aminoglycosides	Mehta et al. (2023)
	Size‐penetration trade‐off	Larger particles carry more drug but penetrate less	Biofilm penetration assays	Keep ≤ 200 nm; stimuli‐responsive shrinkage/disassembly in situ	Dendrimer–AZM clusters that disassemble	Beach et al. (2024)
	Reporting gaps (EE% vs. loading)	Overestimation of clinical feasibility	Publication audits	Mandate both EE% and loading (w/w); mass balance	Journal reporting checklists	Hamdallah et al. (2024)
Models, trials, and regulation	Planktonic/oversimple in vitro tests	Poor prediction of biofilm efficacy	Static plates; Calgary device	Use dynamic/microfluidic models; gradient control; ex vivo tissues	Bioflux, BiofilmChip platforms	Cometta et al. (2024)
	Limited in vivo relevance/time	Chronicity, polymicrobiality undermodeled	Short rodent studies	Longer‐term, site‐matched, polymicrobial models; clinical isolates	CF‐lung alginate bead models	O'Toole et al. (2021)
	Fragmented regulatory pathways	Reclassification with minor tweaks	Case‐by‐case filings	Nano‐specific guidance; standardized characterization/toxicology	Polymer or surface‐modified variants	Desai et al. (2025)
NP‐linked resistance	Adaptive tolerance/resistance selection	Potential cross‐effects on antibiotics	In vitro adaptation studies	Dosing stewardship; combo therapy; rotate mechanisms	ROS‐generating metals with antibiotics	Amaro et al. (2021)
	Dormancy induction in biofilms	Persisters survive; relapse risk	Time‐kill/relapse models	Front‐loaded release + sustained tail; antipersister add‐ons	Nitroxide hybrids; quorum inhibitors	Rao et al. (2021)
Biodistribution and toxicity	Protein corona remodeling	Targeting ligand masking; altered PK/PD	Serum/mucus corona assays	Antifouling coats (PEG/zwitterions); ligand overhang; corona‐aware design	Mucus‐penetrating PEGylated NPs	Pelaz et al. (2017)
	Penetration through ECM	Limited reach to deep layers	Confocal penetration mapping	Charge/size tuning; enzymatic coformulation; ECM‐binding decoys	DNase/Proteinase‐K liposomes	Blanco‐Cabra et al. (2024)
	Clearance/opsonization	Fast RES uptake; subtherapeutic exposure	In vivo PK; organ biodistribution	25–150 nm window; stealth coatings; dosing routes matched to site	Inhaled lung‐targeted nano‐ABX	Y. Zhang et al. (2025)
	ROS/hyperthermia host damage	Oxidative stress; thermal injury	Histology, biomarker panels	Tight energy control; local triggers; dose caps	Photothermal Au; magnetothermal Fe_3_O_4_	Amaral et al. (2025)
	Microbiome disruption and ecology	Dysbiosis; environmental impact	Microbiome sequencing; ecotox	Local delivery; biodegradable carriers; disposal plans	Topical Ag limits; polymer biodegradation	X. Zhang et al. (2024)

Abbreviations: ABX, antibiotic; AgNPs, silver nanoparticles; AZM, azithromycin; CF, cystic fibrosis; DoE, design of experiments; ECM, extracellular matrix; EE%, encapsulation efficiency (%); EtO, evapotranspiration; GMP, good manufacturing practice; NPs, nanoparticles; PD, pharmacodynamic; PEG, poly(ethylene glycol); PEGylated, pegylation; PK, pharmacokinetic; PLGA, poly(lactic‐*co*‐glycolic acid); QbD, quality by design; RES, reticuloendothelial system; ROS, reactive oxygen species; w/w, weight per weight.

## 4 Conclusion

Chronic infections driven by bacterial biofilms represent an escalating challenge for modern healthcare systems. Their intrinsic tolerance to antimicrobial drugs and ability to evade host immune defenses make them particularly difficult to eradicate, often leading to prolonged disease, high treatment costs, and increased morbidity. This reality underscores the pressing need for novel therapeutic approaches capable of overcoming the shortcomings of conventional antibiotics (Salam et al. 2023). Nanotechnology has gained considerable attention as a potential solution, offering innovative strategies to penetrate and disrupt biofilms, enhance drug delivery, and reduce treatment failures. Despite this promise, the journey from preclinical investigation to widespread clinical application remains fraught with obstacles (Campos et al. 2025). Barriers include the complexity and expense of NP synthesis, issues of large‐scale reproducibility, and concerns about long‐term stability and storage that can compromise cost‐effectiveness (Bi et al. 2025). In parallel, the field of biofilm research itself poses inherent challenges: the absence of universally reliable models that faithfully mimic the clinical biofilm environment, the variability of bacterial strains used in studies, and the possibility of microbes evolving resistance even against advanced nanomedicines (Y. Su, Yrastorza, et al. 2022).

Equally important are biological considerations once NPs are introduced into the human body. Biodistribution is not always predictable, and the rapid formation of a biomolecular corona from host fluids can substantially modify NP properties. These interactions can alter drug release profiles, distort pharmacokinetic and pharmacodynamic behavior, and affect clearance pathways, sometimes increasing toxicity or reducing efficacy (Akhter et al. 2021). Taken together, while nanomedicine represents one of the most promising avenues for addressing biofilm‐associated infections, its clinical translation requires overcoming scientific, technical, and regulatory hurdles (Campos et al. 2025). Progress will depend on refining manufacturing processes, improving preclinical biofilm models, anticipating resistance mechanisms, and ensuring safety through rigorous studies of biodistribution and host interactions. Only by addressing these interconnected challenges can nanotechnology fulfill its potential as a transformative tool in the fight against biofilm‐related diseases (Yaraki et al. 2022).

## Author Contributions

**Husni Farah, Munthar Kadhim‐Abosaoda, Hayjaa Mohaisen‐Mousa, S. Renuka Jyothi, Priya Priyadarshini‐Nayak, J. Bethanney Janney, Gurjant Singh, Ashish Singh‐Chauhan, Manoj Kumar Mishra:** conceptualization, writing – original draft, writing – review and editing, supervision.

## Funding

The authors received no specific funding for this work.

## Ethics Statement

The authors have nothing to report.

## Consent

The authors have nothing to report.

## Conflicts of Interest

None declared.

## Data Availability

The authors have nothing to report.
